# Epigenetic Effect of Maternal Methyl-Group Donor Intake on Offspring’s Health and Disease

**DOI:** 10.3390/life12050609

**Published:** 2022-04-19

**Authors:** Szilvia Bokor, Réka A. Vass, Simone Funke, Tibor Ertl, Dénes Molnár

**Affiliations:** 1 Department of Paediatrics, University of Pécs Medical School, H-7624 Pécs, Hungary; molnar.denes@pte.hu; 2 National Laboratory for Human Reproduction, University of Pécs, H-7624 Pécs, Hungary; vass.reka@pte.hu (R.A.V.); funke.simone@pte.hu (S.F.); ertl.tibor@pte.hu (T.E.); 3 Department of Obstetrics and Gynaecology, University of Pécs Medical School, H-7624 Pécs, Hungary

**Keywords:** maternal methyl-group donor intake, epigenetic, DNA methylation, reproductivity, perinatal outcome, non-communicable disease

## Abstract

Maternal exposure to some dietary and environmental factors during embryonic development can affect offspring’s phenotype and, furthermore, the risk of developing diseases later in life. One potential mechanism responsible for this early programming may be the modification of the epigenome, such as DNA methylation. Methyl-group donors are essential for DNA methylation and are shown to have an important role in fetal development and later health. The main goal of the present review is to summarize the available literature data on the epigenetic effect (DNA methylation) of maternal methyl-group donor availability on reproductivity, perinatal outcome, and later health of the offspring. In our literature search, we found evidence for the association between alterations in DNA methylation patterns caused by different maternal methyl-group donor (folate, choline, methionine, betaine) intake and reproductivity, birth weight, neural tube defect, congenital heart defect, cleft lip and palate, brain development, and the development of obesity and associated non-communicable diseases in later life. We can conclude that maternal methyl-group donor availability could affect offspring’s health via alterations in DNA methylation and may be a major link between early environmental exposure and the development of diseases in the offspring. However, still, further studies are necessary to confirm the associations and causal relationships.

## 1. Introduction

Adverse conditions during pregnancy such as suboptimal nutrition of the mater, alcohol consumption, smoking, diabetes, and assisted reproductive technologies (ART) can affect embryonic/fetal epigenetic processes resulting in unfavorable perinatal and long-term health outcomes, such as prematurity, intrauterine growth restriction, increased risk of obesity, metabolic syndrome, and neurodevelopmental disorders [1]. This is the concept of fetal programming that is well confirmed in the literature [2,3]. Epigenetic modifications, such as DNA methylation, are one of the leading underlying mechanisms responsible for health programming [4]. DNA methylation modifies DNA by methylating cytosine bases at the carbon-5′ position in cytosine-guanine (CpG) dinucleotide residues. This methyl-group addition influences the expression of the gene and has been shown to decrease the expression levels of the gene and prevent protein coding. Differential methylation of promoter regions of various genes seems to be associated with early programming. DNA methylation marks on the paternal and maternal genome are globally demethylated shortly after fertilization, which is followed by de novo methylation right before the implantation. During pregnancy, this is a critical window of fetal development where maternal diet can affect the fetal methylome [5].

**Figure 1:**
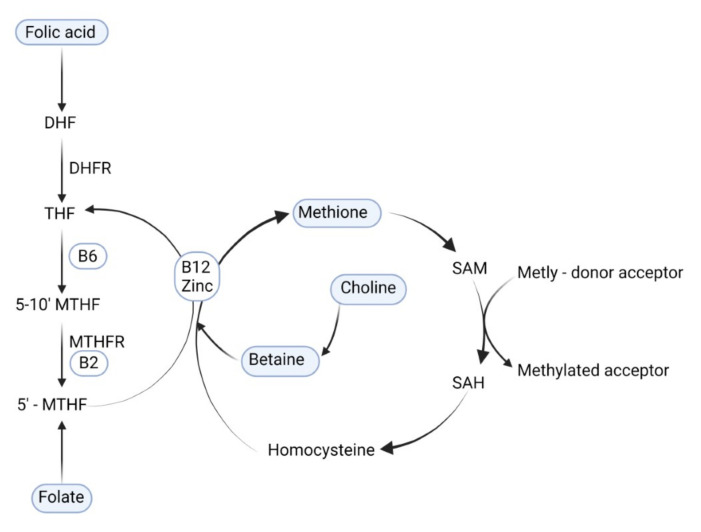
The role of methyl-group donors nutrients in DNA methylation. DHF: dihydrofolate; THF: tetrahydrofolate; B6: vitamin B6; B2: vitamin B2; B12: vitamin B12; DHFR: dihydrofolate reductase; 5-10′ MTHF: 5, 10-methylene tetrahydrofolate; MTHFR: methylenetetrahydrofolate reductase; 5′ MTHF: 5-methyl tetrahydrofolate; SAM: S-adenosylmethionine; SAH: S-adenosylhomocysteine.

One-carbon cycle metabolism nutrients play an important role in more than one hundred sites of the metabolic chain in several biochemical regulation pathways. They are essential for nucleic acid synthesis, DNA methylation, cellular function, metabolism, proliferation, and growth [6,7,8]. Dietary methyl-group donors (folate, folic acid, betaine, choline, and methionine) enter the one-carbon metabolism at different sites but finally are converted to the universal methyl-group donor S-adenosylmethionine (SAM). SAM provides a methyl-group for the methylation of the DNA [9]. Figure 1. In one-carbon metabolism, the majority of methyl groups are derived from choline (60%), methionine (20%), and folate (10–20%) [10].

The deficiency of folate is associated with preterm birth, low birth weight, spontaneous abortion, and other unfavorable birth outcomes such as neural tube defects (NTD) [11,12]. Humans are not able to synthesize folate; it must be consumed by dietary sources (cereals, beans, green leafy vegetables, liver, egg yolks) [11]. In the intestine, dietary folate is absorbed and metabolized to 5-methyl tetrahydrofolate (Figure 1). Folic acid (FA) is a synthetic compound with higher bioavailability than naturally occurring folate but is structurally similar to folate. FA is used in fortified foods and in supplements [13]. Based on the recognized link between fetal neural tube defects and maternal folate status, the WHO approved in 2006 the recommendation that reproductive-aged women should consume 400 μg of folic acid per day from the moment of contemplating pregnancy up to 12 weeks of gestation. In 2015, the WHO recommended 906 nmol/L folate concentrations in red blood cells as optimal to prevent NTDs during the first semester [14]. The benefits of continued FA supplementation in later pregnancy, however, are still under investigation. At the present time, no consistent agreement exists if there is a need to keep high levels of FA intake during the whole pregnancy.

Choline has a role in the lipid-cholesterol metabolism and transport in the structural integrity of cell membranes and in the normal development of the brain [15]. During pregnancy, there is an extra requirement for choline, and this increases the choline demand of the mother, which may exceed the capacity of choline production [16]. New recommendations for adequate intake levels for choline were established recently by The European Food Safety Authority for pregnant women 480 mg and for lactating women 520 mg (European Food Safety Authority, Parma, Italy, 17 August 2016). The main dietary sources of choline are meat, fish, whole grains, eggs, vegetables, and fruit, as well as oils and fats.

Betaine is a methyl donor and an osmolyte. As an osmolyte, betaine protects proteins, enzymes, and cells from environmental stress (e.g., extreme temperature, low water, or high salinity). Betaine participates as a methyl donor in the methionine cycle, primarily in the human kidneys and liver [17]. Betaine can be consumed directly with different food sources (spinach, seafood, and wheat germ) or converted from a choline-containing diet [18]. Methionine is an amino acid required for protein synthesis and is also a key source of methyl groups. Methionine is found in high protein foods such as eggs, meat, fish, and some nuts and cereal grains.

Confirmation that the availability of methyl donors during gestation is essential in order to establish and maintain DNA methylation patterns comes from experiments in the yellow *A^vy^ agouti* mice. Supplementation of the yellow agouti (A*^vy^*) mice pregnant dams with one methyl-group donor nutrients, including methionine, choline, betaine, folic acid, and vitamin B12, resulting in a shift of the fur color in the offspring from yellow to brown [19]. The A*^vy/a^* coat color (yellow, slightly mottled, mottled, heavily mottled, and pseudo-agouti) correlates with the methylation status of A*^vy^* [17]. Furthermore, the *A^vy^* gene predisposes the mice to obesity, hyperinsulinemia, diabetes, susceptibility to hyperplasia, increased somatic growth, and tumorigenesis [20]. The shift to brown fur color was directly due to increased DNA methylation at a cryptic promoter in an intracisternal A particle upstream of the A*^vy^* locus [21,22]. Since then, many animal studies have confirmed the effect of maternal supplementation of methyl-group donors on the epigenome of the offspring, with consequences on health and disease development in later life.

Despite the importance of methyl-group donor nutrients in DNA methylation, only a few human studies have investigated whether maternal methyl-group donor supplementation influences the offspring’s DNA methylation patterns [23,24,25]. Most of the published human studies have primarily investigated only the association between DNA methylation pattern of offspring and maternal methyl-group donor intake without investigating the health and longevity of the offspring or the effect of maternal methyl-group donor intake on the health and longevity of the offspring without investigating DNA methylation patterns.

In the present narrative review, we summarize the available literature data on the epigenetic effect of maternal methyl-group donor intake on reproductivity, the perinatal outcome, and obesity and related diseases. Id est, the present review focuses exclusively on papers investigating the effect of methyl-group donor intake on DNA methylation and phenotype together. The authors are aware that maternal methyl-group donor intake may impact the development of other non-communicable diseases such as respiratory diseases, tumorigenesis, and mental disorders, but the inclusion of these issues would extremely extend the volume of the manuscript and are beyond the scope of the present review.

## 2. Methods

We included animal and human studies where the associations of the three following parameters were investigated together: 1. the DNA methylation pattern of at least one gene of the offspring; 2. data on at least one of the methyl-group donor status or intake of the mother; 3. at least one health outcome of the offspring. Those manuscripts not fulfilling the above-described criteria were excluded. Published literature was retrieved through searches of Medline, PubMed, and the Cochrane Library in November 2021 using appropriate controlled keywords and vocabulary (e.g., folate, folic acid, choline, methionine, betaine, methyl-group donor, one-carbon metabolism, epigenetic, DNA methylation, reproductivity, assisted reproduction technology, birth defects, neural tube defect, cleft lip, congenital heart defect, birth weight, obesity, non-communicable diseases). The detailed descriptions of studies (investigating the epigenetic effect of maternal methyl-group donor intake on offspring’s health and disease) included in the manuscript are summarized in Table S1.

## 3. Epigenetic Effect of Maternal Methyl-Group Donor Intake on Reproductivity

To date, multiple reproductive functions of folate have been published by several studies, and only some functions for the other methyl-group donors. During the second and third trimesters, the requirement for FA is largely increased due to enlarged maternal blood volume and the fast growth of breast and placental tissues [26]. When deficiency of folate occurs, pregnant women are prone to placental abruption, premature spontaneous abortion, stillbirth, preeclampsia, pregnancy hypertension syndrome, and megaloblastic anemia; however, these results are still conflicting [26,27]. It has also been suggested that folate could be related to infertility [10] and may be an important factor in oocyte maturation, the implantation process, and the progression of normal pregnancy [28].

DNA methylation disturbances could explain the link between methyl-group donor intake and reproductivity effects. DNA methylation profiles are highly dynamic during gametogenesis and early embryonic development [29]. In fact, two periods of epigenetic reprogramming across the genome occur within this timeframe [30]. The first period of DNA methylation reprogramming takes place during gametogenesis, where DNA methylation is erased genome-wide and later re-methylated. The DNA remethylation in oocytes and sperms occurs at different developmental times [30]. In females, germ cell DNA methylation patterns are acquired after birth when oocytes enter the growth phase preceding ovulation. In males, the reacquisition of DNA methylation begins in the prenatal gonad [29]. The second period of DNA methylation reprogramming takes place during the preimplantation period after fertilization in the early embryo [30]. During this second wave of reprogramming, there is genome-wide demethylation and re-methylation with the exception of imprinted genes and certain repeat elements, which are not altered [31,32].

Only a few animal studies and even fewer human studies have examined the epigenetic impact of low or high maternal methyl-group donors on developing germ cells and reproductivity. For example, in the study of Ly et al., an exposure to FA supplementation or folate deficiency in female mouse models during early life was reported to have unfavorable effects on their reproductive health in the future and also on their progenies (alterations in DNA methylation and reproductive loss) [33].

### The Epigenetic Role of Maternal Methyl-Group Donor Status in the Perinatal Outcomes of Assisted Reproduction Technology

Growing evidence suggests that although the majority of children conceived through ART are healthy, they are at increased risk for several perinatal complications, birth defects, alterations in later body composition, metabolic and hormonal changes, and rare imprinting disorders [34,35,36,37]. Multiple exposures of ART occur at critical windows of development coinciding with global epigenetic reprogramming [38]. In a recent systematic review of Breton-Larrivée et al., the epigenetic consequences of various ART procedures are summarized [39]. Still, not much is known about the association of epigenetic effect of maternal methyl-group donor availability in ART conceived children and its association with adverse offspring outcomes. Restriction of methyl-group donor nutrients in sheep around the time of conception led to genome-wide epigenetic modifications with long-term implications for the health of the ART conceived offspring [40]. In this study, embryo-donor ewes were provided either methyl-donor-deficient (i.e., deficient in folate, B12, and methionine) or a standard control diet from 8 weeks prior and 6 days after conception by artificial insemination. Following single embryo transfer to normally fed ewes, birthweight or pregnancy rate was not affected, but the offspring later in their life were fatter, insulin resistant, hypertensive, and obtained altered immune responses to antigenic challenges. In 2010, Ménézo et al. also suggested that DNA methylation changes in mammals conceived by ART could result from an altered availability of methyl-donor nutrients [41]. Later, Rahimi et al. showed in a mouse model that moderate dose FA supplementation in ART was associated with advantageous effects, including a decrease in embryonic developmental delay, moderately increased global DNA methylation in placental and embryonic tissues, and reduced variance in DNA methylation at certain imprinting control regions (ICRs) [38]. With our literature search, we did not find published studies in humans on the epigenetic effect of maternal methyl-group donor intake on the health and development of the offspring conceived by ART.

## 4. Epigenetic Effect of Maternal Methyl-Group Donor Intake on Perinatal Outcome

A woman’s nutrition before and during pregnancy may play an important role in normal pregnancy outcomes. In this period, nutritional requirements, including methyl-group donor nutrients (i.e., folate, choline, betaine, and methionine), are higher because of the developing and growing fetus [42,43].

Among the nutrients involved in one-carbon metabolism, maternal folate has been the most extensively studied for its effect on perinatal outcomes. The last three decades of research have shown overwhelming evidence that folate is essential for the health and disease of the offspring. When folate deficiency occurs, the fetus is at risk of intrauterine growth retardation, low birth weight and preterm birth, congenital malformations, in particular, neural tube defects, congenital heart defects, and cleft lip [11,12,26,44,45]. However, most of these results are still conflicting [46]. Variation in consumption of folate could affect the fetal outcome by at least three different mechanisms: perturbation in DNA biosynthesis, toxic accumulation of homocysteine levels, and alteration in methylation reactions [47].

Choline plays a role in the structural integrity of cell membranes, in the lipid-cholesterol metabolism and transport, and in the normal development of the brain [15]. The role of maternal choline intake on fetal development, pregnancy outcomes, and epigenetic programming of post-natal health was recently reviewed [48,49]. Alterations in choline intake could affect the fetal outcomes by four different mechanisms: changes in membrane synthesis, perturbation of acetylcholine biosynthesis, toxic accumulation of homocysteine levels, and alteration in methylation reactions [47].

Betaine plays an essential role in embryo preimplantation, in which it may have a role as an osmolyte and may influence the accurate formation of the neural tube [17,50].

An inappropriate balance of methionine intake can unfavorably influence fetal development and short-term reproductive function through altered homocysteine production or by the alteration of endocrine functions [51].

**Figure 2:**
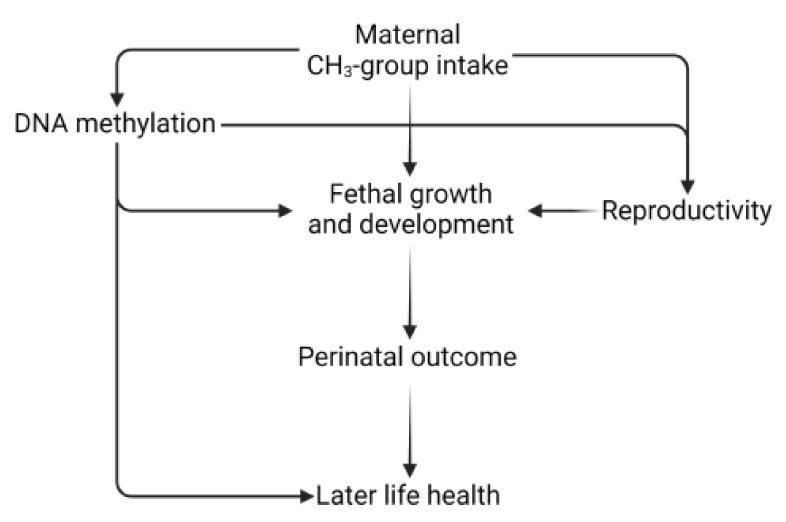
The hypothetical pathways that might link maternal methyl-group donor intake, offspring’s DNA methylation pattern, perinatal health outcomes, and later health hazards.

The associations between maternal methyl-group donor intake and in utero development may be mediated by epigenetic processes. Several investigations were conducted in the field of maternal methyl-group donor intake before and during pregnancy and DNA methylation pattern of the newborn in animals and only some in humans [50,52]. Furthermore, there are some studies where the association between DNA methylation pattern of differently methylated regions (DMRs) and perinatal health outcomes, mainly associated with birth weight, congenital malformations of the offspring, and imprinted disorders, was described [53,54,55]. For example, Cooney et al. [56] showed that maternal dietary methyl-group supplementation with extra folate, choline, betaine, and vitamin B12 increased the methylation of DNA and methylation-dependent epigenetic phenotypes in mammalian offspring. However, comparable findings observed in animals where maternal supplementation of methyl-donor-induced phenotypic changes mediated via DNA methylation are scanty in humans. The hypothetical pathways that might link maternal methyl-group donor intake, offspring’s DNA methylation pattern, and perinatal health outcomes are presented in Figure 2.

In summary, the most investigated perinatal outcomes in association with maternal methyl-group donor intake are fetal growth and congenital malformations such as neural tube defects, brain development, orofacial cleft and palate, and congenital heart defects. Therefore, in this manuscript, we focused our literature search on studies where the epigenetic effect of maternal methyl-group donor intake on the above-mentioned perinatal outcomes was investigated.

### 4.1. Low Birth Weight

In humans, one of the most investigated DNA regions in connection with the epigenetic effect of methyl-group donor intake and birth weight is the insulin-like growth factor 2 (*IGF2*) locus, which covers a ∼150 kb genomic region on human chromosome 11. It contains two imprinted genes, *IGF2* and *H19*. The *IGF2* gene is paternally imprinted, while *H19* is maternally imprinted. If unmethylated, the expression of IGF2 is suppressed, but *H19* is expressed [57]. Studies suggest that *IGF2* promotes the growth, division, and apoptosis of cells in several different tissues. *IGF2* is vital for embryo development. It plays an important role in growth and development before birth [58]. In human individuals exposed to FA supplementation during the periconceptional and prenatal periods, DNA methylation differences of the DMRs regulating the imprinted expression of *IGF2* were shown. Hoyo et al. found an association between methylation variation at *IGF2* DMRs and maternal folic acid use before and during pregnancy [59]. Another study published that supplementation of FA directly impacted the *IGF2* gene methylation status in infants up to 17 months of age. Children of mothers who took FA supplementation had a 4.5% higher methylation of the *IGF2* DMR than children who were not exposed to FA. In this study, an independent, inverse association between *IGF2* DMR methylation and birth weight was also observed [60]. These results may present the plasticity of *IGF2* methylation by periconceptional FA supplementation [60].

Some other studies found an association between different one-carbon nutrient intake and DNA methylation in the region of retrotransposon long interspersed nucleotide element-1 (*LINE-1*), PLAG1-like zinc finger 1 (*ZAC1, PLAGL1*), and maternally expressed gene 3 (MEG3) gene regions in association with low birth weight.

The *LINE-1* plays an important role during embryonic development and can induce birth defects via RNA intermediates [61]. In the mouse model, maternal dietary folate deficiency was described to impair one-carbon metabolism, leading to global DNA and *LINE-1* hypomethylation followed by increased retrotransposition in fetuses, leading to intrauterine growth retardation [61]. A study in humans reported that FA supplementation in pregnant women showed methylation of specific CpG dinucleotides in *LINE-1*. In this study, methylation of *LINE-1* was positively associated with offspring birth weight [62]. In a further study by Fryer et al., CpG methylation patterns in cord blood were associated with plasma homocysteine, birth weight, and LINE-1 methylation, providing further evidence that folate-associated intermediates in the mothers’ diet can affect pregnancy outcomes and the global methylation status of the offspring [63].

The *ZAC1* gene is part of a network of co-regulated imprinted genes (the maternal allele is methylated and consequently not expressed) and is involved in the control of embryonic growth. Loss of methylation at the *ZAC1* DMRs was associated with the development of transient neonatal diabetes mellitus, which is a developmental disorder involving diabetes and growth retardation in the first weeks of post-natal life [25,64]. In the study of Azzi et al., the methylation of the *ZAC1* DMR was positively associated with the estimated weight of the fetus (at 32 weeks of gestation), birth weight, and post-natal weight (at one year of age), and BMI. Furthermore, a maternal diet rich in vitamin B2 (vitamin B2 serves as a cofactor for the enzymes methylenetetrahydrofolate reductase and methionine synthase reductase in one-carbon metabolism) also increased *ZAC1* DMR methylation. In this study, *ZAC1* DMR methylation showed no association with periconceptional or pregnancy FA supplementation. The effects of variation in *ZAC1* methylation were shown only in the second period of pregnancy, and it persisted until at least one year of post-natal age [25].

*MEG3* is an imprinted gene, and it is located on human chromosome 14q32.3. The published association between the methylation status of *MEG3* DMRs and offspring’s health outcomes, such as birth weight, diabetes, diabetes-related diseases, and some cancers, was summarized by Chen et al. [65]. Hoyo et al. observed an inverse relationship between maternal folate levels and DNA methylation for *H19, MEG3, MEG3-IG*, *PEG10/SGCE, NNAT, PLAGL1*, and the *PEG3* DMRs, furthermore positive associations were observed in relation to the *PEG1/MEST* and *IGF2* DMRs. Birth weight was associated with DNA methylation at four DMRs; higher birth weight was associated with higher methylation levels at the *PLAGL1, H19*, and *PEG10/SGCE* DMRs and lower *MEG3* methylation levels suggestive of threshold effects. However, the authors observed statistically significant evidence for mediation of the relationship between birth weight and maternal folate levels only by the *MEG3* DMR [24].

According to the latest review of the Pregnancy and Childhood Consortium, DNA methylation in neonatal blood was associated with birth weight at 914 sites. The difference in birth weight ranged from 183 to 178 g per 10% increase in methylation. Some of the different methylation patterns were also detected in childhood and adolescence but not in adults. Küppers et al. reported an overlap between birthweight-related CpGs and some CpGs that were previously linked to maternal smoking and BMI during pregnancy but not with those associated with folate levels in pregnancy [53].

### 4.2. Cleft Lip and Cleft Palate

The etiology of isolated orofacial clefts is largely unknown, and it is suspected to be etiologically heterogeneous with both non-genetic and genetic risk factors [66]. Maternal use of folic acid-containing multivitamin supplements in early pregnancy has been associated with decreased risk of cleft lip and cleft palate (CL/P) [67]. Several observations support the hypothesis that epigenetic alterations may be one of the underlying mechanisms by which folic acid may reduce CL/P risk [45]. Sharp et al. [68] described differentially methylated regions that may be characteristic of different subtypes of clefts. Alvizi et al. showed in a CL/P case-control study methylation changes at individual CpGs and proposed that DNA methylation alterations may interact with genetic risk factors to increase penetrance [69]. Gonseth et al. have evaluated whole genomic DNA methylation levels using newborn bloodspots and found 28 significantly hypomethylated CpGs mapped to *WNT9B, VTRNA2–1, LHX8*, and *MIR140*. The results showed that periconceptional folic acid supplementation could reduce the risk of orofacial clefts in offspring [45].

### 4.3. Congenital Heart Defects

Congenital heart defects (CHD) are thought to have multifactorial etiology, but the main causes are largely unknown. In the past years, several studies reported on the possible contribution of DNA methylation abnormalities to CHD [54,70]. The epigenetic roles of maternal nutrition in congenital heart disease have been well summarized by Radha O Joshi et al. [44]. Despite these studies, in our literature search, we found only one study where the epigenetic effect of maternal methyl-group donor intake was investigated in relation to CHD outcome. Gonzales-Pena et al. recently reported a relationship between maternal dietary folic acid consumption and lower methylation status of Axis inhibitor 1 (*AXIN1*) gene and higher methylation status of T-box transcription factor 20 (*TBX20*) genes associated with a ventricular septal defect in children [71] Both genes have been previously shown to be related to CHD [72].

### 4.4. Neural Tube Defects

Neural tube defects (NTDs) are presumably caused by a combination of environmental factors and genetic defects. Several epidemiological studies have indicated that lower folate concentrations during pregnancy are related to an increased risk of NTDs [11,12,46].

Epigenetic modifications may be one of the underlying mechanisms by which folate deficiency may increase NTD risk [73,74,75]. The study of Wang et al. showed an association between hypomethylation of genomic DNA and *LINE-1* and an increased risk of NTDs. Maternal plasma vitamin B-12 insufficiency was associated with NTDs, and no significant correlation could be established between *LINE-1* methylation and maternal folic acid and homocysteine [76]. These results were partially confirmed in the study of Chang et al., where *LINE-1* hypomethylation was found in embryonic tissues from NTD mice [77]. In this study, the authors hypothesized that intracellular folate reduction leads to *LINE-1* hypomethylation, which restrains the transformation of embryonic stem cells into the embryoid body, finally inducing NTDs. Both *LINE-1* and global DNA methylation levels were decreased in nervous tissues from NTD fetuses, and the levels of folate and vitamin B-12 showed a similar decline pattern in maternal plasma [77].

Rochtus et al. reviewed the association between one-carbon metabolism, DNA methylation, and NTDs [74]. The authors concluded that folate deficiency may increase NTD risk by decreasing DNA methylation, but to date, human studies on the association of NTDs and epigenetic modifications vary widely in study design in terms of analyzing different clinical subtypes of NTDs, using different DNA methylation quantification assays and using DNA isolated from diverse types of tissues. Findings of global DNA hypomethylation and *LINE-1* hypomethylation suggest that epigenetic alterations may disrupt neural tube closure. However, the research [72] does not support a linear relation between red blood cell folate concentration and DNA methylation.

### 4.5. Brain Development and Cognitive Functions

Alterations in one-carbon metabolism during embryonic development can affect the methylation signatures of neural cells during brain development, resulting in neurodevelopmental aberrations [78]. Data so far indicate that the alteration in the micronutrient consumption of such as folate, choline, methionine, betaine, and B vitamins may alter phenotypes through methylation-related mechanisms by altering gene or global methylation during critical windows of brain development.

Studies have demonstrated that prenatal micronutrient changes in pregnant rats can alter global methylation in the brains of Wistar rat offspring. These studies demonstrated a state of hypermethylation in the cortex of the adult offspring in response to an imbalance in maternal levels of VitB12 and folic acid [79].

Caffrey and colleagues investigated the consequence of maternal folic acid supplementation on the cognitive functions of the offspring. The analysis demonstrated significant changes in the methylation status of candidate genes (*TBM46*, *LINE-1*, *IGF2*, *APC2*, *PEG3*, *OPCML*, *BDNF*, *GRIN3B,* and *GRB10*) related to brain development in the blood samples of newborns. These results showed a link between maternal folate intake and neurodevelopment of the offspring [80].

## 5. Epigenetic Effect of Maternal One-Carbon Metabolism Nutrients on Offspring Obesity and Associated Non-Communicable Diseases

In recent years the concept of obesity as an “epigenetic disease” has emerged [81]. According to this new theory, exosomes, such as maternal nutrition during early pregnancy, leave a “nutritional imprint” in the newborn with long-term effects on the promotion of obesity and related diseases in adulthood [81,82].

Maternal diets low in methyl donors led to agouti gene hypomethylation in mice [19], which resulted in increased somatic growth, obesity, diabetes, hyperinsulinemia, and tumorigenesis in the offspring [18]. Inadequate maternal one-carbon donor intake during critical periods of early embryonic development has been linked to lower DNA methylation at the agouti locus [83], predisposing to adult obesity, diabetes, and cancer [84]. The importance of the changes in DNA methylation occurring in the third trimester of pregnancy was emphasized in the manuscript of Kantake et al., where the promoter methylation levels of *IGF1* were shown to be significantly reduced in infants with IUGR compared to term infants [85]. These results suggest that the critical time window for programming of later body growth may occur not only at the early embryonic stage.

A decreased concentration of leptin and leptin promoter hypermethylation in offspring was observed after a maternal methyl-supplemented diet in rats. Impaired post-natal growth was found in both offspring sexes, and males exhibited increased long-term body weight gain [86].

The study of the Maternal Nutrition and Offspring’s Epigenome cohort of mother-infant pairs showed that maternal periconceptional methyl-donor intake (folic acid, folate, betaine) may epigenetically alter offspring’s genes related to growth: *IGF2*, metabolism: Retinoid X receptor, alpha and appetite control: Leptin gene (*LEP*) [50].

In infants aged 6 months, *LEP* gene methylation in buccal cells was inversely associated with prepregnancy folate intake and second-trimester folate and betaine intake [50]. The hormone leptin is produced by the *LEP* gene, which is involved in regulating appetite; therefore, changes in its expression may predispose to obesity and associated non-communicable diseases in adulthood.

Higher intake of maternal choline (480 vs. 930 mg/d) in the third trimester yielded higher placental promoter methylation of the cortisol-regulating genes, corticotropin-releasing hormone, and glucocorticoid receptor and 33% lower cord plasma cortisol [87]. These results suggest that gestational choline intake may influence offspring stress reactivity by DNA methylation. An elevated response to stress increases the risk of depression, type 2 diabetes mellitus, immunological disorders, and hypertension later in life [88].

## 6. Conclusions

Maternal methyl-group donor availability may be associated with fetal growth and development and with different perinatal outcomes such as birth weight, congenital malformations, and later health hazards. There is mounting evidence to demonstrate that offspring’s DNA methylation pattern may be affected by maternal methyl-group donor (folate, betaine, choline, and methionine) intake during pregnancy. Despite these research results, only a few human studies were conducted, where differences in offspring’s DNA methylation, the amount of maternal methyl-group donor intake, and offspring’s health were investigated in the same study.

In our literature search, we found evidence for the association between alterations in DNA methylation patterns caused by different maternal methyl-group donor (folate, choline, methionine, betaine) intake and reproductivity, birth weight, neural tube defect, congenital heart defect, cleft lip and cleft palate, brain development and the development of obesity and associated non-communicable diseases in later life.

These results indicate that the alteration in the consumption of methyl-group donors during pregnancy may alter offspring’s phenotypes through a methylation-related mechanism by altering global or gene methylation during critical periods of fetal development. The results further indicate that the adequate maternal methyl-group donor intake may be important, not only in the first semester of pregnancy (for which maternal intake recommendations exist) but also during the whole pregnancy to prevent offspring from non-communicable diseases in later life.

As gene expression is regulated not only by DNA methylation but also by other epigenetic mechanisms such as histone modifications, micro RNAs, and 3D chromatin organization, still further studies are required to better understand the interactions and crosstalk between the different epigenetic mechanisms.

## Data Availability

Not applicable.

## Supplementary Materials

The following supporting information can be downloaded at: https://www.mdpi.com/article/10.3390/life12050609/s1, Table S1: Description of studies investigating the epigenetic effect of maternal methyl-group donor intake on offspring’s health and disease.

## References

[1] Rubini E., Baijens I.M.M., Horánszky A., Schoenmakers S., Sinclair K.D., Zana M., Dinnyés A., Steegers-Theunissen R.P.M., Rousian M. (2021). Maternal One-Carbon Metabolism during the Periconceptional Period and Human Foetal Brain Growth: A Systematic Review. Genes.

[2] Moster D., Lie R.T., Markestad T. (2008). Long-Term Medical and Social Consequences of Preterm Birth. N. Engl. J. Med..

[3] Lillycrop K.A., Burdge G.C. (2015). Maternal Diet as a Modifier of Offspring Epigenetics. J. Dev. Orig. Health Dis..

[4] McGee M., Bainbridge S., Fontaine-Bisson B. (2018). A Crucial Role for Maternal Dietary Methyl Donor Intake in Epigenetic Programming and Fetal Growth Outcomes. Nutr. Rev..

[5] Faulk C., Dolinoy D.C. (2011). Timing Is Everything. Epigenetics.

[6] Kalhan S.C., Marczewski S.E. (2012). Methionine, Homocysteine, One Carbon Metabolism and Fetal Growth. Rev. Endocr. Metab. Disord..

[7] Radziejewska A., Chmurzynska A. (2019). Folate and Choline Absorption and Uptake: Their Role in Fetal Development. Biochimie.

[8] Rush E.C., Katre P., Yajnik C.S. (2014). Vitamin B12: One Carbon Metabolism, Fetal Growth and Programming for Chronic Disease. Eur. J. Clin. Nutr..

[9] Anderson O.S., Sant K.E., Dolinoy D.C. (2012). Nutrition and Epigenetics: An Interplay of Dietary Methyl Donors, One-Carbon Metabolism and DNA Methylation. J. Nutr. Biochem..

[10] Niculescu M.D., Zeisel S.H. (2002). Diet, Methyl Donors and DNA Methylation: Interactions between Dietary Folate, Methionine and Choline. J. Nutr..

[11] Fekete K., Berti C., Cetin I., Hermoso M., Koletzko B.v., Decsi T. (2010). Perinatal Folate Supply: Relevance in Health Outcome Parameters. Matern. Child Nutr..

[12] Tamura T., Picciano M.F. (2006). Folate and Human Reproduction. Am. J. Clin. Nutr..

[13] Barua S., Kuizon S., Junaid M.A. (2014). Folic Acid Supplementation in Pregnancy and Implications in Health and Disease. J. Biomed. Sci..

[14] Cordero A.M., Crider K.S., Rogers L.M., Cannon M.J., Berry R.J. (2015). Optimal Serum and Red Blood Cell Folate Concentrations in Women of Reproductive Age for Prevention of Neural Tube Defects: World Health Organization Guidelines. MMWR Morb. Mortal. Wkly. Rep..

[15] Ueland P.M. (2011). Choline and Betaine in Health and Disease. J. Inherit. Metab. Dis..

[16] Zeisel S.H., Mar M.H., Zhou Z., da Costa K.A. (1995). Pregnancy and Lactation Are Associated with Diminished Concentrations of Choline and Its Metabolites in Rat Liver. J. Nutr..

[17] Craig S.A. (2004). Betaine in Human Nutrition. Am. J. Clin. Nutr..

[18] Jiang X., West A.A., Caudill M.A. (2014). Maternal Choline Supplementation: A Nutritional Approach for Improving Offspring Health?. Trends Endocrinol. Metab..

[19] Wolff G.L., Kodell R.L., Moore S.R., Cooney C.A. (1998). Maternal Epigenetics and Methyl Supplements Affect Agouti Gene Expression in Avy/a Mice. FASEB J..

[20] Miltenberger R.J., Mynatt R.L., Wilkinson J.E., Woychik R.P. (1997). The Role of the Agouti Gene in the Yellow Obese Syndrome. J. Nutr..

[21] Yi P., Melnyk S., Pogribna M., Pogribny I.P., Hine R.J., James S.J. (2000). Increase in Plasma Homocysteine Associated with Parallel Increases in Plasma S-Adenosylhomocysteine and Lymphocyte DNA Hypomethylation. J. Biol. Chem..

[22] Waterland R.A. (2003). Do Maternal Methyl Supplements in Mice Affect DNA Methylation of Offspring?. J. Nutr..

[23] Ba Y., Yu H., Liu F., Geng X., Zhu C., Zhu Q., Zheng T., Ma S., Wang G., Li Z. (2011). Relationship of Folate, Vitamin B12 and Methylation of Insulin-like Growth Factor-II in Maternal and Cord Blood. Eur. J. Clin. Nutr..

[24] Hoyo C., Daltveit A.K., Iversen E., Benjamin-Neelon S.E., Fuemmeler B., Schildkraut J., Murtha A.P., Overcash F., Vidal A.C., Wang F. (2014). Erythrocyte Folate Concentrations, CpG Methylation at Genomically Imprinted Domains, and Birth Weight in a Multiethnic Newborn Cohort. Epigenetics.

[25] Azzi S., Sas T.C., Koudou Y., le Bouc Y., Souberbielle J.-C., Dargent-Molina P., Netchine I., Charles M.-A. (2014). Degree of Methylation of *ZAC1* (*PLAGL1*) Is Associated with Prenatal and Post-Natal Growth in Healthy Infants of the EDEN Mother Child Cohort. Epigenetics.

[26] Cai S., Quan S., Yang G., Ye Q., Chen M., Yu H., Wang G., Wang Y., Zeng X., Qiao S. (2021). One Carbon Metabolism and Mammalian Pregnancy Outcomes. Mol. Nutr. Food Res..

[27] Lassi Z.S., Salam R.A., Haider B.A., Bhutta Z.A. (2013). Folic Acid Supplementation during Pregnancy for Maternal Health and Pregnancy Outcomes. Cochrane Database Syst. Rev..

[28] Ebisch I.M.W., Thomas C.M.G., Peters W.H.M., Braat D.D.M., Steegers-Theunissen R.P.M. (2007). The Importance of Folate, Zinc and Antioxidants in the Pathogenesis and Prevention of Subfertility. Hum. Reprod. Update.

[29] Smallwood S.A., Kelsey G. (2012). De Novo DNA Methylation: A Germ Cell Perspective. Trends Genet..

[30] Messerschmidt D.M., Knowles B.B., Solter D. (2014). DNA Methylation Dynamics during Epigenetic Reprogramming in the Germline and Preimplantation Embryos. Genes Dev..

[31] Lane N., Dean W., Erhardt S., Hajkova P., Surani A., Walter J., Reik W. (2003). Resistance of IAPs to Methylation Reprogramming May Provide a Mechanism for Epigenetic Inheritance in the Mouse. Genesis.

[32] Brandeis M., Kafri T., Ariel M., Chaillet J.R., McCarrey J., Razin A., Cedar H. (1993). The Ontogeny of Allele-Specific Methylation Associated with Imprinted Genes in the Mouse. EMBO J..

[33] Ly L., Chan D., Landry M., Angle C., Martel J., Trasler J. (2020). Impact of Mothers’ Early Life Exposure to Low or High Folate on Progeny Outcome and DNA Methylation Patterns. Environ. Epigenetics.

[34] Hoorsan H., Mirmiran P., Chaichian S., Moradi Y., Hoorsan R., Jesmi F. (2017). Congenital Malformations in Infants of Mothers Undergoing Assisted Reproductive Technologies: A Systematic Review and Meta-Analysis Study. J. Prev. Med. Public Health.

[35] Cortessis V.K., Azadian M., Buxbaum J., Sanogo F., Song A.Y., Sriprasert I., Wei P.C., Yu J., Chung K., Siegmund K.D. (2018). Comprehensive Meta-Analysis Reveals Association between Multiple Imprinting Disorders and Conception by Assisted Reproductive Technology. J. Assist. Reprod. Genet..

[36] Heber M.F., Ptak G.E. (2021). The Effects of Assisted Reproduction Technologies on Metabolic Health and Disease†. Biol. Reprod..

[37] Guo X.-Y., Liu X.-M., Jin L., Wang T.-T., Ullah K., Sheng J.-Z., Huang H.-F. (2017). Cardiovascular and Metabolic Profiles of Offspring Conceived by Assisted Reproductive Technologies: A Systematic Review and Meta-Analysis. Fertil. Steril..

[38] Rahimi S., Martel J., Karahan G., Angle C., Behan N.A., Chan D., MacFarlane A.J., Trasler J.M. (2019). Moderate Maternal Folic Acid Supplementation Ameliorates Adverse Embryonic and Epigenetic Outcomes Associated with Assisted Reproduction in a Mouse Model. Hum. Reprod..

[39] Breton-Larrivée M., Elder E., McGraw S. (2019). DNA Methylation, Environmental Exposures and Early Embryo Development. Anim. Reprod..

[40] Sinclair K.D., Allegrucci C., Singh R., Gardner D.S., Sebastian S., Bispham J., Thurston A., Huntley J.F., Rees W.D., Maloney C.A. (2007). DNA Methylation, Insulin Resistance, and Blood Pressure in Offspring Determined by Maternal Periconceptional B Vitamin and Methionine Status. Proc. Natl. Acad. Sci. USA.

[41] Menezo Y., Elder K., Benkhalifa M., Dale B. (2010). DNA Methylation and Gene Expression in IVF. Reprod. Biomed. Online.

[42] Pauwels S., Duca R., Devlieger R., Freson K., Straetmans D., van Herck E., Huybrechts I., Koppen G., Godderis L. (2016). Maternal Methyl-Group Donor Intake and Global DNA (Hydroxy)Methylation before and during Pregnancy. Nutrients.

[43] Dominguez-Salas P., Cox S.E., Prentice A.M., Hennig B.J., Moore S.E. (2012). Maternal Nutritional Status, C_1_ Metabolism and Offspring DNA Methylation: A Review of Current Evidence in Human Subjects. Proc. Nutr. Soc..

[44] Joshi R.O., Chellappan S., Kukshal P. (2020). Exploring the Role of Maternal Nutritional Epigenetics in Congenital Heart Disease. Curr. Dev. Nutr..

[45] Gonseth S., Shaw G.M., Roy R., Segal M.R., Asrani K., Rine J., Wiemels J., Marini N.J. (2019). Epigenomic Profiling of Newborns with Isolated Orofacial Clefts Reveals Widespread DNA Methylation Changes and Implicates Metastable Epiallele Regions in Disease Risk. Epigenetics.

[46] De-Regil L.M., Peña-Rosas J.P., Fernández-Gaxiola A.C., Rayco-Solon P. (2015). Effects and Safety of Periconceptional Oral Folate Supplementation for Preventing Birth Defects. Cochrane Database Syst. Rev..

[47] Zeisel S.H. (2009). Importance of Methyl Donors during Reproduction. Am. J. Clin. Nutr..

[48] Korsmo H.W., Jiang X., Caudill M.A. (2019). Choline: Exploring the Growing Science on Its Benefits for Moms and Babies. Nutrients.

[49] Zeisel S. (2017). Choline, Other Methyl-Donors and Epigenetics. Nutrients.

[50] Pauwels S., Ghosh M., Duca R.C., Bekaert B., Freson K., Huybrechts I., Langie S.A.S., Koppen G., Devlieger R., Godderis L. (2017). Maternal Intake of Methyl-Group Donors Affects DNA Methylation of Metabolic Genes in Infants. Clin. Epigenetics.

[51] Rees W.D., Wilson F.A., Maloney C.A. (2006). Sulfur Amino Acid Metabolism in Pregnancy: The Impact of Methionine in the Maternal Diet. J. Nutr..

[52] Joubert B.R., den Dekker H.T., Felix J.F., Bohlin J., Ligthart S., Beckett E., Tiemeier H., van Meurs J.B., Uitterlinden A.G., Hofman A. (2016). Maternal Plasma Folate Impacts Differential DNA Methylation in an Epigenome-Wide Meta-Analysis of Newborns. Nat. Commun..

[53] Küpers L.K., Monnereau C., Sharp G.C., Yousefi P., Salas L.A., Ghantous A., Page C.M., Reese S.E., Wilcox A.J., Czamara D. (2019). Meta-Analysis of Epigenome-Wide Association Studies in Neonates Reveals Widespread Differential DNA Methylation Associated with Birthweight. Nat. Commun..

[54] Cao J., Wu Q., Huang Y., Wang L., Su Z., Ye H. (2021). The Role of DNA Methylation in Syndromic and Non-Syndromic Congenital Heart Disease. Clin. Epigenetics.

[55] Elhamamsy A.R. (2017). Role of DNA Methylation in Imprinting Disorders: An Updated Review. J. Assist. Reprod. Genet..

[56] Cooney C.A., Dave A.A., Wolff G.L. (2002). Maternal Methyl Supplements in Mice Affect Epigenetic Variation and DNA Methylation of Offspring. J. Nutr..

[57] Bartolomei M.S., Webber A.L., Brunkow M.E., Tilghman S.M. (1993). Epigenetic Mechanisms Underlying the Imprinting of the Mouse H19 Gene. Genes Dev..

[58] (2013). Brenner’s Encyclopedia of Genetics. Brenner’s Encyclopedia of Genetics.

[59] Hoyo C., Murtha A.P., Schildkraut J.M., Jirtle R.L., Demark-Wahnefried W., Forman M.R., Iversen E.S., Kurtzberg J., Overcash F., Huang Z. (2011). Methylation Variation at *IGF2* Differentially Methylated Regions and Maternal Folic Acid Use before and during Pregnancy. Epigenetics.

[60] Steegers-Theunissen R.P., Obermann-Borst S.A., Kremer D., Lindemans J., Siebel C., Steegers E.A., Slagboom P.E., Heijmans B.T. (2009). Periconceptional Maternal Folic Acid Use of 400 Μg per Day Is Related to Increased Methylation of the IGF2 Gene in the Very Young Child. PLoS ONE.

[61] Li B., Chang S., Liu C., Zhang M., Zhang L., Liang L., Li R., Wang X., Qin C., Zhang T. (2019). Low Maternal Dietary Folate Alters Retrotranspose by Methylation Regulation in Intrauterine Growth Retardation (IUGR) Fetuses in a Mouse Model. Med Sci. Monit..

[62] Fryer A.A., Nafee T.M., Ismail K.M.K., Carroll W.D., Emes R.D., Farrell W.E. (2009). LINE-1 DNA Methylation Is Inversely Correlated with Cord Plasma Homocysteine in Man: A Preliminary Study. Epigenetics.

[63] Fryer A.A., Emes R.D., Ismail K.M.K., Haworth K.E., Mein C., Carroll W.D., Farrell W.E. (2011). Quantitative, High-Resolution Epigenetic Profiling of CpG Loci Identifies Associations with Cord Blood Plasma Homocysteine and Birth Weight in Humans. Epigenetics.

[64] Arima T. (2001). A Conserved Imprinting Control Region at the HYMAI/ZAC Domain Is Implicated in Transient Neonatal Diabetes Mellitus. Hum. Mol. Genet..

[65] Chen C., Jiang Y., Yan T., Chen Y., Yang M., Lv M., Xi F., Lu J., Zhao B., Luo Q. (2021). Placental Maternally Expressed Gene 3 Differentially Methylated Region Methylation Profile Is Associated with Maternal Glucose Concentration and Newborn Birthweight. J. Diabetes Investig..

[66] Dixon M.J., Marazita M.L., Beaty T.H., Murray J.C. (2011). Cleft Lip and Palate: Understanding Genetic and Environmental Influences. Nat. Rev. Genet..

[67] Jahanbin A., Shadkam E., Miri H.H., Shirazi A.S., Abtahi M. (2018). Maternal Folic Acid Supplementation and the Risk of Oral Clefts in Offspring. J. Craniofacial Surg..

[68] Sharp G.C., Ho K., Davies A., Stergiakouli E., Humphries K., McArdle W., Sandy J., Davey Smith G., Lewis S.J., Relton C.L. (2017). Distinct DNA Methylation Profiles in Subtypes of Orofacial Cleft. Clin. Epigenetics.

[69] Alvizi L., Ke X., Brito L.A., Seselgyte R., Moore G.E., Stanier P., Passos-Bueno M.R. (2017). Differential Methylation Is Associated with Non-Syndromic Cleft Lip and Palate and Contributes to Penetrance Effects. Sci. Rep..

[70] Serra-Juhé C., Cuscó I., Homs A., Flores R., Torán N., Pérez-Jurado L.A. (2015). DNA Methylation Abnormalities in Congenital Heart Disease. Epigenetics.

[71] González-Peña S.M., Calvo-Anguiano G., Martínez-de-Villarreal L.E., Ancer-Rodríguez P.R., Lugo-Trampe J.J., Saldivar-Rodríguez D., Hernández-Almaguer M.D., Calzada-Dávila M., Guerrero-Orjuela L.S., Campos-Acevedo L.D. (2021). Maternal Folic Acid Intake and Methylation Status of Genes Associated with Ventricular Septal Defects in Children: Case–Control Study. Nutrients.

[72] Hernández-Almaguer M.D., Calvo-Anguiano G., Cerda-Flores R.M., Salinas-Torres V.M., Orozco-Galicia F., Glenn E., García-Guerra J., Sánchez-Cortés G., Lugo-Trampe J., Martínez-Garza L.E. (2019). Genetic Variants at the Rs4720169 Locus of *TBX20* and the Rs12921862 Locus of *AXIN1* May Increase the Risk of Congenital Heart Defects in the Mexican Population: A Pilot Study. Genet. Test. Mol. Biomark..

[73] Lu X.-L., Wang L., Chang S.-Y., Shangguan S.-F., Wang Z., Wu L.-H., Zou J.-Z., Xiao P., Li R., Bao Y.-H. (2016). Sonic Hedgehog Signaling Affected by Promoter Hypermethylation Induces Aberrant Gli2 Expression in Spina Bifida. Mol. Neurobiol..

[74] Rochtus A., Jansen K., Geet C., Freson K. (2015). Nutri-Epigenomic Studies Related to Neural Tube Defects: Does Folate Affect Neural Tube Closure Via Changes in DNA Methylation?. Mini-Rev. Med. Chem..

[75] Wang L., Shangguan S., Xin Y., Chang S., Wang Z., Lu X., Wu L., Niu B., Zhang T. (2017). Folate Deficiency Disturbs Hsa-Let-7 g Level through Methylation Regulation in Neural Tube Defects. J. Cell. Mol. Med..

[76] Wang L., Wang F., Guan J., Le J., Wu L., Zou J., Zhao H., Pei L., Zheng X., Zhang T. (2010). Relation between Hypomethylation of Long Interspersed Nucleotide Elements and Risk of Neural Tube Defects. Am. J. Clin. Nutr..

[77] Chang S., Wang L., Guan Y., Shangguan S., Du Q., Wang Y., Zhang T., Zhang Y. (2013). Long Interspersed Nucleotide Element-1 Hypomethylation in Folate-Deficient Mouse Embryonic Stem Cells. J. Cell. Biochem..

[78] Mastrototaro G., Zaghi M., Sessa A. (2017). Epigenetic Mistakes in Neurodevelopmental Disorders. J. Mol. Neurosci..

[79] Sable P., Randhir K., Kale A., Chavan-Gautam P., Joshi S. (2015). Maternal Micronutrients and Brain Global Methylation Patterns in the Offspring. Nutr. Neurosci..

[80] Caffrey A., Irwin R.E., McNulty H., Strain J.J., Lees-Murdock D.J., McNulty B.A., Ward M., Walsh C.P., Pentieva K. (2018). Gene-Specific DNA Methylation in Newborns in Response to Folic Acid Supplementation during the Second and Third Trimesters of Pregnancy: Epigenetic Analysis from a Randomized Controlled Trial. Am. J. Clin. Nutr..

[81] Navarro E., Funtikova A.N., Fíto M., Schröder H. (2017). Prenatal Nutrition and the Risk of Adult Obesity: Long-Term Effects of Nutrition on Epigenetic Mechanisms Regulating Gene Expression. J. Nutr. Biochem..

[82] Barker D.J. (1990). The Fetal and Infant Origins of Adult Disease. BMJ.

[83] Waterland R.A., Jirtle R.L. (2003). Transposable Elements: Targets for Early Nutritional Effects on Epigenetic Gene Regulation. Mol. Cell. Biol..

[84] Fall C. (2009). Maternal Nutrition: Effects on Health in the next Generation. Indian J. Med. Res..

[85] Kantake M., Ikeda N., Nakaoka H., Ohkawa N., Tanaka T., Miyabayashi K., Shoji H., Shimizu T. (2020). IGF1 Gene Is Epigenetically Activated in Preterm Infants with Intrauterine Growth Restriction. Clin. Epigenetics.

[86] Giudicelli F., Brabant A.-L., Grit I., Parnet P., Amarger V. (2013). Excess of Methyl Donor in the Perinatal Period Reduces Postnatal Leptin Secretion in Rat and Interacts with the Effect of Protein Content in Diet. PLoS ONE.

[87] Jiang X., Yan J., West A.A., Perry C.A., Malysheva O.v., Devapatla S., Pressman E., Vermeylen F., Caudill M.A. (2012). Maternal Choline Intake Alters the Epigenetic State of Fetal Cortisol-regulating Genes in Humans. FASEB J..

[88] Xiong F., Zhang L. (2013). Role of the Hypothalamic–Pituitary–Adrenal Axis in Developmental Programming of Health and Disease. Front. Neuroendocrinol..

