# Supplementary material for: Epigenetic Effect of Maternal Methyl-Group Donor Intake on Offspring’s Health and Disease
Source: Life (Basel). 2022 Apr 19;12(5):609. doi: 10.3390/life12050609 (PMC9145757; doi:10.3390/life12050609)
Supplement: Supplementary file 1 [file life-12-00609-s001.zip › life-1594929-supplementary.pdf]

life

MDPI

Supplementary Materials

Epigenetic Effect of Maternal Methyl-Group Donor Intake on Offspring's Health and Disease

Szilvia Bokor [1,2,*], Réka A. Vass [2,3], Simone Funke [2,3], Tibor Ertl [2,3] and Dénes Molnár [1,2]

1 Department of Paediatrics, University of Pécs Medical School, H-7624 Pécs, Hungary; molnar.denes@pte.hu
2 National Laboratory for Human Reproduction, University of Pécs, H-7624 Pécs, Hungary; vass.reka@pte.hu (R.A.V.); funke.simone@pte.hu (S.F.); ertl.tibor@pte.hu (T.E.)
3 Department of Obstetrics and Gynaecology, University of Pécs Medical School, H-7624 Pécs, Hungary
* Correspondence: bokor.szilvia@pte.hu

Table S1. Description of studies investigating the epigenetic effect of maternal methyl-group donor intake on offspring's health and disease.

Study	Supplementations and/or measured parameters	Time of supplementation/ determination of maternal plasma methyl-group donor	DNA methylation pattern	Observed phenotype/outcome
		**Animal studies**		
Waterland R.A et al. *Molecular and Cellular Biology* **2003**, *23*, 5293–5300 [85]	In mouse (a/a females): folic acid, vitamin B12, choline chloride, and anhydrous betaine	2 weeks before mating and throughout pregnancy and lactation	Increased CpG methylation at the Avy locus	No effect on litter size or offspring body weight at age 21 days.<br>Coat color distribution of A vy /a offspring shifted toward the brown (pseudoagouti) phenotype
Sinclair K.D. et al. *Proceedings of the National Academy of Sciences* **2007**, *104*, 19351–19356 [40]	In sheep: „methyl-deficient'" (MD) diet, reduced vitamin B12, folate, and methionine	In Embryo-donor ewes from 8 weeks prior and 6 days after conception by artificial insemination. (Day-6 blastocysts recovered after artificial insemination were transferred singly to normally fed surrogate ewes.)	In fetal liver of ART conceived offspring at gestational day 90 methylation status of 1,400 CpG sites were analyzed.<br>71 loci were identified that varied from the entire control group in only a single MD individual. Further 57 loci were altered in two or more MD animals.<br>88% of these loci were unmethylated or hypomethylated relative to controls. The remaining loci were hypermethylated.	-No effects on pregnancy establishment or birth weight.<br>-Adult offspring (22-23 months of age) were heavier and fatter, elicited altered immune responses to antigenic challenge, were insulin-resistant, and had elevated blood pressure.
Chang S et al. *Journal of*	Mouse embryonic stem cells (ESCs):	For 6, 12, and 18 days	At day 18, most CpG sites of LINE-1 showed significant	No effect on mouse ESCs proliferation and differentiation

Cellular Biochemistry **2013**, *114*, 1549–1558. [79]	ESCs were cultured in folate-free (FF) medium, folate-deficient (FD) medium, and folate-normal (FN) medium		hypomethylation in FF and FD compared with that in FN	
Giudicelli F et al. *PLoS ONE* **2013**, *8*, e68268 [88]	In rats: methionine, folic acid, choline, betaine, zinc, Vit B12 supplementation	21 to 28 days before mating, and throughout gestation and lactation	DNA methylation of 16 CpG sites of the leptin gene promoter was analyzed. Significantly higher DNA methylation was observed for CPG sites: 3, 4, 6, 7, 10, 11, 12, 13, 14 and 16.	Plasma glucose was increased (18%), plasma leptin levels in 21 days old rats was reduced (-40%)
Rahimi S. et al. *Human Reproduction* **2019**, *34*, 851–862. [38]	In mice: Three groups according to folic acid supplementation: 1: a folic acid control diet (CD) containing the recommended level of folate for rodents, 2: a 4-fold folic acid-supplemented diet (4FASD) 3: a 10-fold folic acid-supplemented diet (10FASD).	Diet for 6 weeks prior to ART and throughout gestation.	In both placentas and embryos (at midgestation) the 4FASD decreased methylation variance at the Kcnq1ot1 and H19 ICRs compared to the CD group. Methylation variance for Snrpn was increased by 10FASD in the placenta and decreased by 4FASD in the embryo. Peg1 methylation variance was not affected in a major way by either dose of folic acid.	-No significant differences in fertilisation rates and in vitro development rates were observed between diet groups.<br>-4FASD in ART was associated with decrease in embryonic developmental delay.<br>-Folic acid supplementation did not significantly affected the overall rate of embryonic abnormalities.
**Human studies**				
Steegers-Theunissen R.P. et al. *PLoS ONE* **2009**, *4*, e7845 [60]	Human study: Folic acid supplementation N= 120 children (of whom 86 mothers had used and 34 had not used folic acid periconceptionally)	400 mg from at least 4 weeks before until 8 weeks after conception	Children of mothers who used folic acid had 4.5% higher methylation of the IGF2 DMR in whole blood samples at the age of 12 and 18 months.	Inverse independent association between IGF2 DMR methylation and birth weight.
Wang L. et al. *The American Journal of Clinical Nutrition* **2010**, *91*, 1359–1367. [77]	Nervous tissue from NTD (neural tube defect) and control samples: Control and case subjects from Stillborn and Medical abortions. Maternal plasma concentrations of folic acid, vitamin B-12, and	Just before the abortion.	Methylation levels of genomic DNA and LINE-1 decreased significantly in the neural tissue of NTD samples.	-NTDs increased with decreasing levels of LINE-1 methylation, with an odds ratio of 5.246 (95% CI: 1.519, 18.124; P = 0.009) for the lowest quartile compared with the highest quartile.<br>-No significant correlation was found between global methylation levels and maternal plasma concentrations of folate, vitamin B-12, or homocysteine.

	homocysteine. (All pregnancies lacked preconception folic acid supplementation.) N=48 NTD and N=49 control samples			
Jiang et al. *The FASEB Journal* **2012** [89]	Choline supplementation of 480 mg/day (n=12) or 930 mg/day N=24	During the third trimester	Higher placental methylation of: -global DNA -corticotropin releasing hormone promoter -glucocorticoid receptor promoter Lower cord blood leukocyte methylation of: - corticotropin releasing hormone - glucocorticoid receptor No methylation changes: -IGF2, IL10, and LEP	33% lower cord plasma cortisol
Azzi S. et al. *Epigenetics* **2014**, *9*, 338–345. [64]	Folic acid, Vitamin B2, B6 and B12 intake of the mother (Food frequency questionnaire). N=378	Use of dietary supplements within three months prior to pregnancy and the use of dietary supplements during the last 3 months of gestation.	Vitamin B2 positively correlated with the ZAC1 DMR methylation index prior pregnancy and in the last 3 months of pregnancy, respectively. The ZAC1 DMR methylation index was not significantly associated with dietary folic acid supplementation.	There was a non-significant correlation between methylation index and birth weight. Neither birth weight, length at birth, length SDS at one year of age, or C-peptide concentration in cord serum correlated with the methylation index of the ZAC1 DMR. At one year of age, the ZAC1 DMR methylation index correlated positively with weight and BMI Z scores.
Hoyo C. et al. *Epigenetics* **2014**, *9*, 1120–1130. [24]	Erythrocyte folate concentrations were measured from maternal venous blood. Study participants were enrolled as part of the Newborn Epigenetics Study. N=496.	In the first trimester.	Higher birth weight was associated with higher DNA methylation levels at the PEG10/SGCE, PLAGL1 and H19 DMRs, and lower methylation levels at the MEG3 and NNAT DMRs. An inverse relationship between maternal folate levels and DNA methylation for H19, MEG3 MEG3-IG, PEG10/SGCE, NNAT, PLAGL1 and the PEG3 DMRs, and positive associations in relation to the PEG1/MEST and IGF2 was found.	DNA methylation at the PLAGL1, SGCE, DLK1/MEG3 and IGF2/H19 DMRs was associated with maternal folate levels and also birth weight, suggestive of threshold effects. MEG3 DMR methylation mediated the association between maternal folate levels and birth weight (P =0.06)
González-Peña S.M. et	In a case-control study of children	Determination of Maternal Dietary	AXIN1 displayed a lower methylation status, while TBX20	No maternal folic acid supplementation was a risk

al. *Nutrients* **2021**, *13*, 2071 [73]	with ventricular septal defects (VSD) and healthy children: Folic acid intake of the mother (Food frequency questionnaire focusing on FA intake from food sources and the consumption of supplements containing FA) VSD patients: N=16 Control patients: N=32	Intake of Folic Acid: before and during pregnancy. 400 mcg/day was considered the cut-off point of FA intake.	displayed a higher methylation status in VSD children compared to control children. Lower methylation status of AXIN1 genes and higher methylation status of TBX20 genes is associated with maternal folic acid supplementation.	factor for VSD in children (OR = 3.909, 95% CI = 2.348–6.508). A low dietary folic acid intake (<400 mcg/day) in the studied women did not relate to a risk of VSD in their children (OR = 1.571, 95% CI = 0.409–6.040).
